# Correction to “Identification of a mutation in *TNRC18* in a patient with clinical features of Fazio–Londe disease”

**DOI:** 10.1002/ccr3.8545

**Published:** 2024-02-20

Khani M, Shamshiri H, Nafissi S, Salehi N, Moazzeni H, Taheri H, Elahi E. Identification of a mutation in *TNRC18* in a patient with clinical features of Fazio–Londe disease. Clin Case Rep. 2024; 12:e8394. *Clin Case Rep*. 2024;12:e8394.

Description of error:

Under the AUTHOR CONTRIBUTIONS section, the name and contribution of one of the authors, Hosein Shamshiri, was not included, and his contribution was mistakenly attributed to Marzieh Kahani. The text as printed is “**Marzieh Khani:** recruitment of patient and family members, exome data analysis, mutation screening and segregation analysis in families by Sanger sequencing, and contribution to writing of manuscript; **Marzieh Khani:** and **Shahriar Nafissi:** patient identification and provision of clinical data; **Najmeh Salehi:** Prediction of three dimensional structure of TNRC18; **Hamidreza Moazzeni:** data analysis; **Hanieh Taheri:** mutation screening and segregation analysis in families by Sanger sequencing; **Elahe Elahi:** design and supervision of the research, and writing the manuscript. All authors approved the final version of the manuscript.”. It should be changed to:

“**Marzieh Khani:** recruitment of patient and family members, exome data analysis, mutation screening and segregation analysis in families by Sanger sequencing, and contribution to writing of manuscript; **Hosein Shamshiri:** and **Shahriar Nafissi:** patient identification and provision of clinical data; **Najmeh Salehi:** prediction of three dimensional structure of TNRC18; **Hamidreza Moazzeni:** data analysis; **Hanieh Taheri:** mutation screening and segregation analysis in families by Sanger sequencing; **Elahe Elahi:** design and supervision of the research, and writing the manuscript. All authors approved the final version of the manuscript.”

